# Mitochondrial pathway and endoplasmic reticulum stress participate in the photosensitizing effectiveness of AE‐PDT in MG63 cells

**DOI:** 10.1002/cam4.895

**Published:** 2016-10-03

**Authors:** Kai‐Ting Li, Qing Chen, Da‐Wu Wang, Qin‐Qin Duan, Si Tian, Juan‐Wen He, Yun‐Sheng Ou, Ding‐Qun Bai

**Affiliations:** ^1^ Department of RehabilitationThe First Affiliated Hospital of Chongqing Medical UniversityChongqingChina; ^2^ Department of GastroenterologyChinese Medicine Hospital of LongquanChengduChina; ^3^ Department of OrthopedicsThe First Affiliated Hospital of Chongqing Medical UniversityChongqingChina

**Keywords:** Aloe emodin, endoplasmic reticulum, mitochondrial, osteosarcoma, photodynamic therapy

## Abstract

Photodynamic therapy (PDT) is a promising treatment in cancer therapy, with a photosensitizer activated by visible light. Aloe‐emodin (AE) is a promising photosensitive agent. In this study, the photosensitizing effects and possible mechanisms of AE‐PDT in MG63 cells were evaluated. The efficiency of AE‐PDT was analyzed by MTT assay. The mode of cell death was investigated by Hoechst 33,342 staining and flow cytometer. The intracellular distribution of AE was detected with confocal microscopy. The formation of reactive oxygen species (ROS) was detected by DCFH‐DA. The mitochondrial membrane potential (MMP) was measured by Rhodamine 123. The expression of proteins including cytochrome c, caspase‐3, ‐9, and ‐12, CHOP and GRP78 was detected by western blot. Apoptosis is the primary mode of cell death in our study, which occurs in a manner of depending on AE concentration and irradiation dose. Confocal microscopy showed that AE was primarily localized on the mitochondria and endoplasmic reticulum (ER) of MG63 cells. AE‐PDT resulted in rapid increases of intracellular ROS production, which reached a peak at 2 h, followed by declining of mitochondrial membrane potential, releasing of cytochrome c from mitochondria into the cytoplasm, and up‐regulation of caspase‐3, ‐9, and ‐12, CHOP and GRP78. These results suggest that death of MG63 cells induced by AE‐PDT is triggered by ROS. Meanwhile, Mitochondria and ER serve as the subcellular targets, which are responsible for AE‐PDT‐induced death of MG63 cells.

## Introduction

Osteosarcoma is one of the most common malignant tumors of the bone 1. Surgery, radiotherapy, and high‐dose chemotherapy have improved survival rate over the past several decades 2. However, the 5‐year survival rates are still no more than 60%. Drug resistance and occurrence of second malignancies remain serious problems 3. Thus, there is an urgent requirement to develop novel approaches to deal with osteosarcoma 4.

Photodynamic therapy (PDT) is an anticancer modality utilizing the generation of singlet oxygen and other reactive oxygen species (ROS), after irradiating photosensitizer with visible light 5, 6. Aloe‐emodin (AE) is an active component from the root and rhizome of Rheum palmatum, which has been reported as a novel photosensitizer and played important role in many kind of carcinomas 7, 8, 9. Our study is going to explore the effect of AE‐PDT on osteosarcoma cell line MG63 and the possible mechanisms.

## Materials and Methods

### Photosensitizer, chemicals, and cell line

Aloe‐emodin (C_34_H_36_N_4_O_3_, 95% of purity) was purchased from Jiangxi Tiangong Technology (Jiangxi, China). A stock solution (100 mmol/L) was made in dimethyl sulfoxide (DMSO), filtered with 0.2 *μ*m PTFE syringe filter (Alltech Association, Inc., Deerfield, IL), then stored in the dark at −20°C. Fetal bovine serum (FBS), DMEM, antibiotics penicillin‐streptomycin‐neomycin (PSN) and trypsin were supplied by Gibco (Grand‐Island, NY). Caspase‐3 and cytochrome c were purchased from CST (Danvers, MA), caspase‐9, caspase‐12 and C/EBP homologous protein (CHOP) were purchased from Immunoway Biotechnology (Newark, DE), GRP78 and *β*‐actin were supplied from Santa Cruz Biotechnology (Santa Cruz, CA). MTT, Hoechst 33342, Rhodamine 123 were obtained from Sigma (St Louis, MA). Annexin V‐ PI apoptosis kit was from Keygen Biotech (Nanjing, Jiangsu, China). The light‐emitting diode (LED) light was purchased from Chongqingjingyu Laser Biological Company (Chongqing, China). MG63 cells were obtained from the American Type Culture Collection (ATCC) and cultured in DMEM supplemented with 10% FBS and antibiotics. Cell cultures were incubated at 37°C in a humidified 5% CO_2_ incubator. Cells in growth period were used for the next experiments.

### PDT protocol

Cells were inoculated into cell culture plates and placed into an incubator for 24 h. Then the cells were divided into four groups—A: 0.01% DMSO, treated as blank control; B: pure AE group; C: pure light group; D: AE‐PDT group. The steps below were conducted in the dark. Group A and C were cultured with DMEM medium free of FBS. Group B and D were incubated with DMEM medium free of FBS, but with AE (10 *μ*mol/L). Six hours later, the medium was replaced by a culture medium with FBS (10%). Cells in group C and D were exposed to LED light (430 nm, 40 mW/cm^2^) for 30–120 sec to obtain an energy density of 1.2–4.8 J/cm^2^, respectively. After irradiation, the cells were recultivated in an incubator according to the time point required.

### Cytotoxicity assay

After 24 h of PDT, the cytotoxicity was assessed by the MTT assay. The medium was removed and the cells were washed twice with PBS gently. MTT dilution was added to each well and then the plate was softly mixed and placed back to the incubator for 4 h. The optical density (OD) was obtained by an iEMS Analyzer at wavelengths of 570 nm. Then the percentage of cytotoxicity was calculated according to the following equation: cytotoxicity (%) = (OD control group − OD treatment group)/OD control group × 100%.

### Measurement of ROS

Cellular ROS contents were measured by DCFH‐DA (Invitrogen, Paisley, UK) staining. Briefly, MG 63 cells (2 × 10^5^ cells/plate) were grown on 6‐well plates for 24 h. Groups dividing and the incubation methods were the same as depicted in the PDT protocol. Cells in the AE‐PDT group were then cultured with 10 *μ*mol/L DCFH‐DA solution, lacking LED light for 10 min, and then irradiated. Thereafter followed immediately, the cells were detected, using 488 nm for excitation and 525 nm for emission at different time points post‐irradiation.

### Apoptosis measured by flow cytometer

Apoptotic cells were analyzed with an Annexin V/PI apoptosis kit, using flow cytometry. The cells (2 × 10^5^ cells/well) were inoculated into 6‐well plates for 24 h. Six hours after treatment, the MG 63 cells were harvested and stained with Annexin V‐FITC and PI (50 mg/mL) according to the manual, then analyzed by Flow Cytometer (Becton Dickinson, San Jose, CA) immediately. The percentage of cells in the different regions was calculated for comparison.

### Nuclear staining

MG63 cells (1 × 10^4^ cells/plate) were grown on 24‐well plates for 24 h. The dividing and the incubation and methods were the same as depicted above. After 12 h of PDT, the cells were washed three times with PBS and fixed with 4% paraformaldehyde for 10 min. Subsequently, the fixed cells were washed thrice with PBS and stained with Hoechst 33342 (2 *μ*g/mL) for 20 min, then observed with fluorescence microscope (Olympus 1X71; Olympus, Tokyo, Japan). The condensation and fragmentation of nuclei were considered to be representative of apoptosis.

### Subcellular localization of AE

During the last 30 min of AE incubation, cells were co‐loaded with Mito‐Tracker green or ER‐Tracker green, at a final concentration of 500 nmol/L, to tag the mitochondria and ER, respectively. An overlapped fluorescence from organelle probe and AE was observed with confocal microscopy (TCS SP5, Leica, Heidelberg, Baden‐Wurttemberg state, German).

### Mitochondrial membrane potential

Rhodamine 123 was used to measure MMP. In brief, suspended the cells of each group with 1 mL PBS and loaded with 1 *μ*mol/L rhodamine 123 for 30 min. Flow cytometry (Becton Dickinson, San Jose, CA) was used to observe the alteration of MMP.

### Western blotting analysis

Western blotting was carried out to observe the protein expressions level. Briefly, at various time points after PDT, cells in AE‐PDT group and control groups were collected and lysed using radio‐immunoprecipitation assay (RIPA) buffer (Kaiji Bio Co., Nanjing, China). The following steps were just as we depicted in our previous study 26.

## Results

### Cytotoxicity of AE‐PDT in the MG63 cells

**Figure 1:**
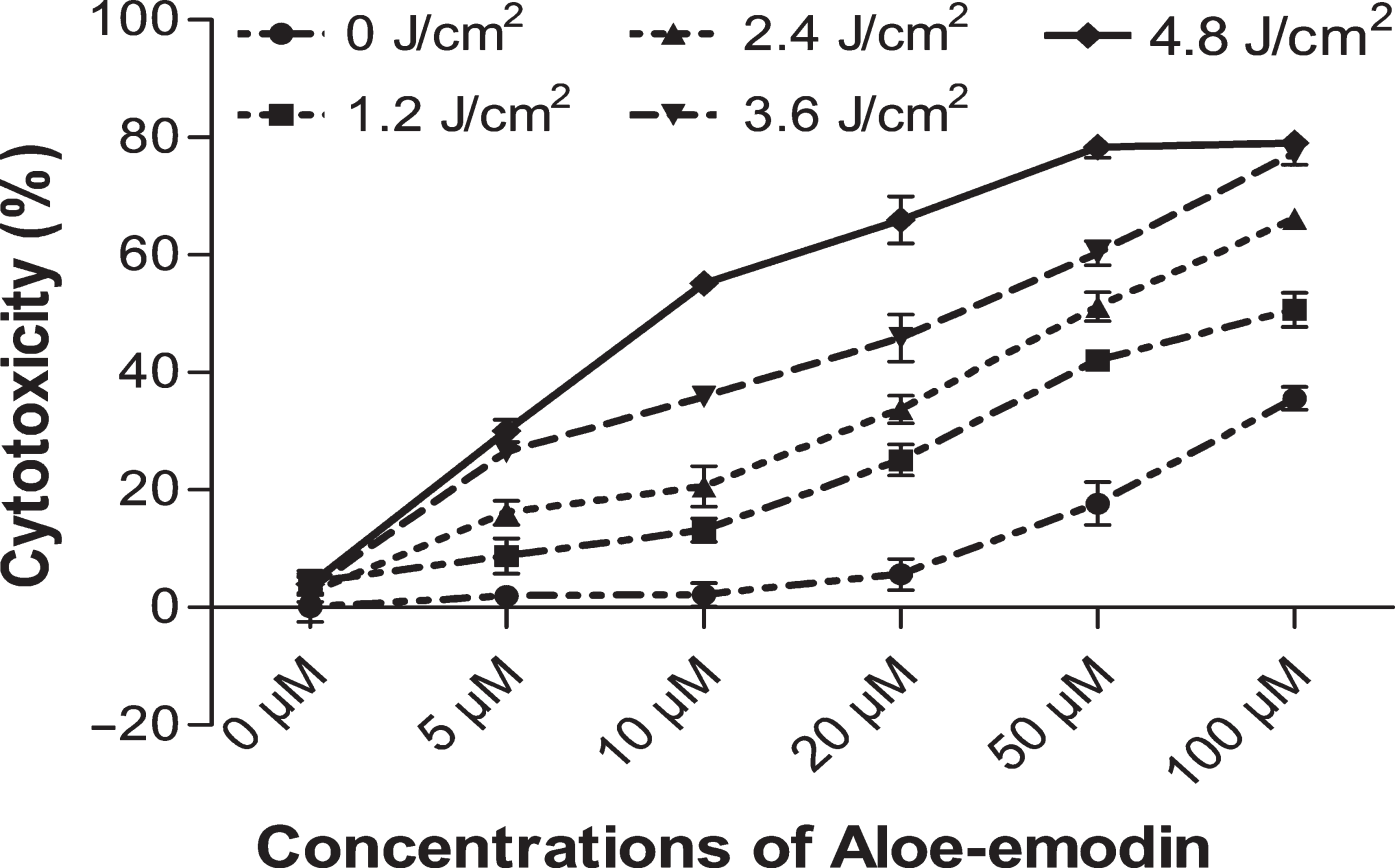
The cytotoxicity of AE‐PDT on human MG63 cells. Drug‐ and light‐dose‐dependent effect on cell cytotoxicity after incubation with Aloe‐emodin for 6 h, followed by irradiation. Data correspond to mean values ± SD from at least three different experiments.

The cytotoxic effect of AE‐ PDT at varied concentrations and energy densities are shown in Figure 1, from which drug‐ and light‐dose–response curves were observed. The results showed that there was no significant dark cytotoxicity when the concentration of AE was lower than 20 *μ*mol/L, but there was apparent cytotoxicity when the concentration was over 50 *μ*mol/L. The results are consistent with the former finding 10, 11. Apparently, AE‐PDT on MG63 cells got an IC50 at 10 *μ*mol/L and 4.8 J/cm^2^, as shown in Figure 1. Therefore, AE concentration of 10 *μ*mol/L and energy density of PDT at 4.8 J/cm^2^ were selected for our next experiments.

### Apoptosis and necrosis of MG63 cells induced by AE‐PDT

**Figure 2:**
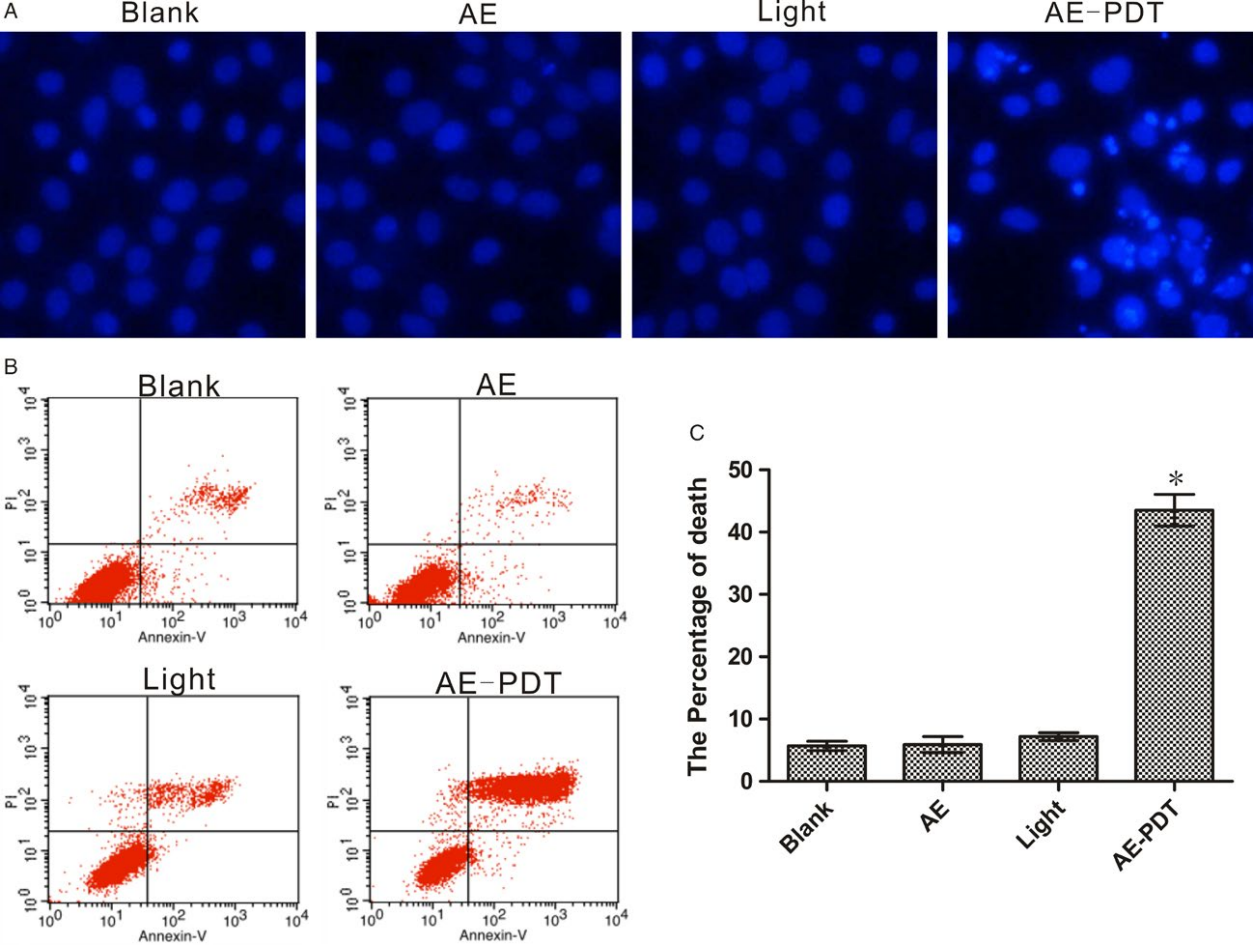
AE‐PDT‐induced death on MG63 cells. (A) Cell apoptosis revealed by Hoechst 33,342 staining of fragmented nuclei of apoptotic cells. (B) Quantified analysis of the extent and mode of cell death percentage by flow cytometer. *P < 0.01, AE‐PDT group versus the three control groups. Values are mean ± SD of three independent determinations.

Cells of AE‐PDT group and control groups (pure AE group, pure light group, and the blank control group) were stained by Hoechst 33,342. Marked morphological changes were observed at 12 h post‐PDT. Density increasing, condensation, coagulation, and fragmentation of the nuclear chromatin were observed, as well as typical apoptotic bodies revealed in the AE‐PDT group, as shown in Figure 2A. While in the other control groups, the nucleus demonstrated intactly rounded/elliptical, no cell apoptosis was observed.

Annexin V/PI staining was utilized to assess the percentage of the apoptotic and necrotic cell induced by AE‐PDT. Data showed that the MG‐63 cells in AE‐PDT group exhibited an apparent increase in both early apoptosis and late apoptosis/necrosis, while the differences in controls were not significant (Fig. 2B and C).

### Subcellular localization of AE

**Figure 3:**
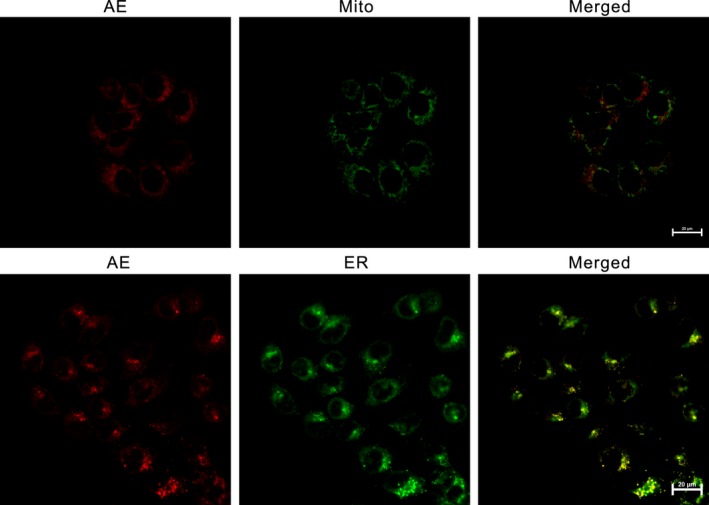
Detected subcellular localization of Aloe‐emodin (AE) by confocal microscopy. Red fluorescent corresponds to AE, green fluorescent shows Mito‐Tracker‐stained mitochondria or ER‐Tracker‐stained endoplasmic reticulum, yellow fluorescent indicates colocalization of red and green fluorescence.

Mito‐tracker and ER‐tracker were used in this study to unfold the subcellular localization of AE in the MG63 cells. Overlapping regions (yellow) were regarded as the subcellular localization of AE. The results showed that the fluorescence images of the mitochondria probe as well as the ER probe were partially overlapping with the AE (Fig. 3).

### Formation of ROS

**Figure 4:**
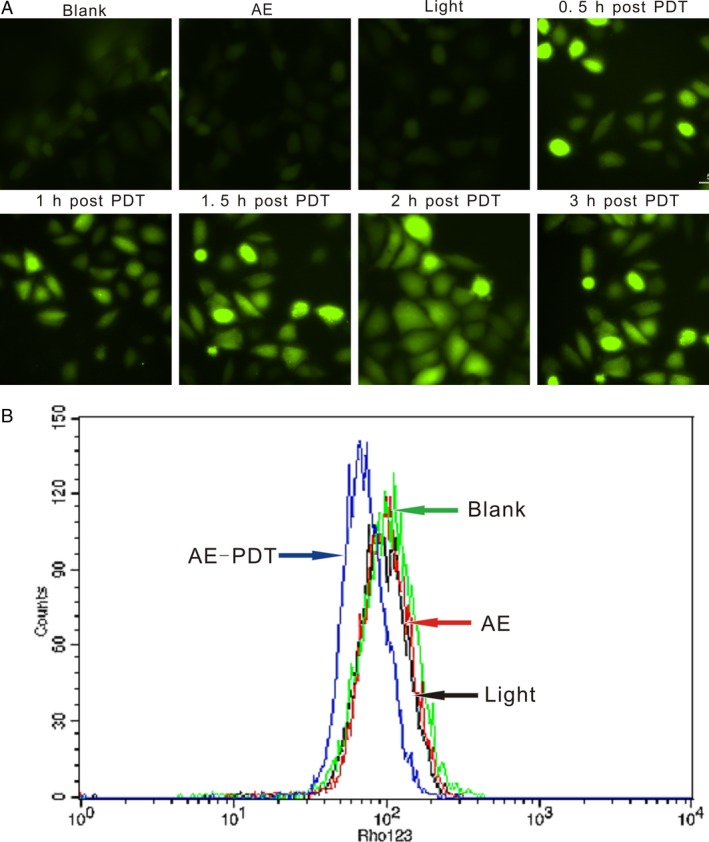
(A) Formation of reactive oxygen species (ROS) was detected by DCFH‐ DA staining. (B) Collapse of mitochondrial membrane potential was detected by Rhodamine 123.

In our experiment, ROS formation initiated by AE‐PDT was monitored. The production of general ROS dramatically increased at 30 min after AE‐PDT. Thereafter, the intensity peaked at 2 h, but attenuated at 3 h following PDT (Fig. 4A).

### Decline of mitochondrial membrane potential

Data from Flow cytometric with Rhodamine 123 staining have showed that there were significant changes of MMP in the MG63 cells 3 h after LED irradiation, and the decline of MMP in AE‐PDT group was observed. However, the differences in control groups were not significant (Fig. 4B).

### Effect of AE‐PDT on mitochondrial‐related protein expression

**Figure 5:**
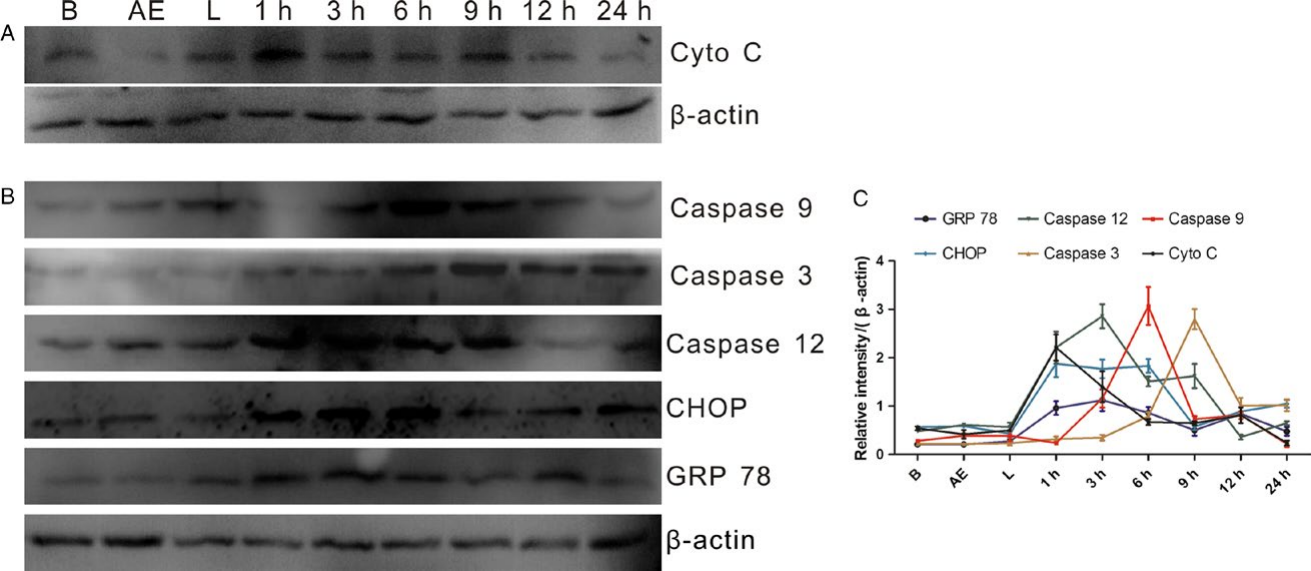
The proteins Expression of Cytochrome c, Caspase 9, 12, 3, CHOP and GRP 78. (A) Effect of Aloe‐emodin (AE) on mitochondrial‐related protein expression; (B) Effect of AE‐PDT on ER stress‐related protein expression. (C) Quantitative analysis of protein expression according to panels (A and B). The vertical axis represents the protein expression relative to *β*‐actin. Data represent means ± SDs (*n* = 3). B means blank control group, AE means single AE group, L means single light group, 1–24 h means 1–24 h post‐PDT.

We have found that AE co‐located on mitochondrial in MG63 cells, so we detected whether the related proteins were activated after AE‐PDT. As shown in Figure 5, the amount of cytosolic cytochrome c was significantly increased at 1 h after PDT. In addition, we found that the expression of caspase‐9 and caspase‐3 demonstrated an early increase and a late decrease pattern, with the peak at 6 and 9 h after PDT, respectively.

### Effect of AE‐PDT on ER stress‐related protein expression

At the same time, we found that AE co‐located on the ER too. Therefore, we detected the expression of ER stress‐related proteins. Data showed that the expression of caspase‐12, Grp78, and CHOP was upregulated after AE‐PDT (Fig. 5), which revealed that AE‐PDT exerted ER stress on MG63 cells.

## Discussion

Aloe‐emodin is a novel anthraquinone derivative extracted from traditional Chinese medicinal plants. Previous studies have demonstrated that AE have antitumor effects with dose‐dependent cytotoxicity 7, 8, 9, 11, 12. However, the genotoxicity limits its application in large dosage 13. Fortunately, AE has been found to be one photosensitizer, which displayed stronger anticancer effect than 5‐aminolevulinic acids with a low concentration 14. Lee et al. 14 have demonstrated that the cytoskeletal alteration was potentially involved in anoikis induced by AE‐PDT in human lung carcinoma H460 cells. Our previous study also demonstrated that AE‐PDT produced significant photocytotoxicity and induced apoptosis in tumors 15, 16. However, the molecular mechanism of AE‐PDT is still not completely clear.

In this study, we also confirmed the marked photocytotoxicity of AE to MG63 cells. As shown by Figure 1, cytotoxicity of AE‐PDT occurred in a manner dependent on concentration and irradiation dose. Furthermore, apoptosis remained the principal mode of MG63 cell death. At the same time, we found these results were consistent with the changes in intracellular ROS, which was produced rapidly and significantly after AE‐PDT (Fig. 4). These results indicate that the cytotoxicity of MG63 cell after PDT is due to the generation of intracellular ROS.

Because of its short life and limited diffusion distance of ROS, the primary site of photodamage coincides with the subcellular localization of the sensitizer 17. Mitochondrial, ER, lysosme, and golgiosome have been reported to be chosen by some sensitizers as subcellular localizations, which is largely dependent on the photosensitizer characteristics as well as the cell type 18. As a new photosensitizer, the subcellular localization of AE was seldom depicted previously. However, in this current study, AE was found to localize mainly in the mitochondria as well as the ER, which prompted us to further examine mitochondrial and ER stress‐associated apoptosis (Fig. 3).

Mitochondria play a significant role in supplying cell energy and regulating cell apoptosis and are considered to be an important target organelle during the PDT 19, 20, 21. Generally, PDT induces high levels of ROS, which have proved to be effective in disrupting the mitochondrial permeability transition pore (MPTP),resulting in immediate collapse of mitochondrial membrane potential (MMP). Following the collapse of the MMP, apoptotic factors (such as cytochrome c) are released and triggers the mitochondrial apoptosis cascade. At last, cell death occurred 22, 23, 24, 25. In our study, a rapid decline of MMP was observed after PDT, which was followed by the release of cytochrome c, caspase‐9, and caspase‐3. We further found that the peak expression of caspase‐9 was observed at 6 h post‐PDT, while caspase‐3 peaked around 9–12 h after PDT (Fig. 5), then, followed with a decreasing trend. The results might because of that caspase proteins were cleaved into small fragments during the apoptotic process.

Endoplasmic reticulum is a cellular organelle that has essential roles in multiple cellular processes and has a great cross‐talk with mitochondria. To seek the effect of ER stress on apoptosis of MG63 cells induced by AE‐PDT, the protein expression of caspase‐12, CHOP, and GRP78 were monitored. All the three factors experienced essential components in ER‐induced apoptosis, and they were considered to be ER stress markers as well as the key regulators of ER stress response signaling 11, 27, 28, 29. In this study, the activation of caspasew‐12, CHOP and GRP78 shared the same response pattern, we observed the overexpression of caspase‐12 after AE‐PDT with peak expression at 3 h, CHOP with peak expression at 6–12 h (Fig. 5), while GRP78 with peak expression at 6 h. Taken together, these results suggested the initiation of the ER‐stress‐specific cascade of apoptosis.

In conclusion, AE‐PDT triggers death of MG63 cells mainly by provoked mitochondrial damage as well as the ER stress, which was supported by the main localization of AE in the mitochondria and ER of MG63 cells.

## Conflict of Interest

We declare that we have no conflict of interest in relation to this article.
